# Association study identified biologically relevant receptor genes with synergistic functions in celiac disease

**DOI:** 10.1038/s41598-019-50120-4

**Published:** 2019-09-25

**Authors:** Pratibha Banerjee, Sandilya Bhagavatula, Ajit Sood, Vandana Midha, B. K. Thelma, Sabyasachi Senapati

**Affiliations:** 1 Department of Human Genetics and Molecular Medicine, School of Health Sciences, Central University of Punjab, Bathinda, Punjab India; 2 Department of Gastroenterology, Dayanand Medical College & Hospital, Ludhiana, Punjab India; 3 Department of Medicine, Dayanand Medical College & Hospital, Ludhiana, Punjab India; 4 Department of Genetics, University of Delhi South Campus, New Delhi, India

**Keywords:** Genome-wide association studies, Coeliac disease

## Abstract

Receptors are essential mediators of cellular physiology, which facilitate molecular and cellular cross-talk with the environment. Nearly 20% of the all known celiac disease (CD) genes are receptors by function. We hypothesized that novel biologically relevant susceptibility receptor genes act in synergy in CD pathogenesis. We attempted to identify novel receptor genes in CD by re-analyzing published Illumina Immunochip dense genotype data for a north Indian and  a European (Dutch) cohort. North Indian dataset was screened for 269 known receptor genes. Association statistics for SNPs were considered with minor allele frequency >15% and association P ≤ 0.005 to attend desired study power. Identified markers were tested for cross-ethnic replication in a European CD dataset. Markers were analyzed *in-silico* to explain their functional significance in CD. Six novel SNPs from *MOG* (rs29231, p = 1.21e-11), *GABBR1* (rs3025643, p = 1.60e-7), *OR2H2* (rs1233388, p = 0.0002), *ABCF1* (rs9262119, p = 0.0005), *ADRA1A* (rs10102024, p = 0.003), and *ACVR2A* (rs7560426, p = 0.004) were identified in north Indians, of which three genes namely, *GABBR1* (rs3025643, p = 5.38e-8), *OR2H2* (rs1233388, p = 3.29e-5) and *ABCF1* (rs9262119, p = 0.0002) were replicated in Dutch. Tissue specific functional annotation, potential epigenetic regulation, co-expression, protein-protein interaction and pathway enrichment analyses indicated differential expression and synergistic function of key genes that could alter cellular homeostasis, ubiquitination mediated phagosome pathway and cellular protein processing to contribute for CD. At present multiple therapeutic compounds/drugs are available targeting *GABBR1* and *ADRA1A*, which could be tested for their effectiveness against CD in controlled drug trials.

## Introduction

Celiac disease (CD) is a chronic autoimmune disease triggered by gluten protein in genetically susceptible individuals. The diagnosis of CD shows an increase in intraepithelial lymphocytes and altered crypt:villus ratio^1^. Although, prevalence of CD varies from 0.5% to 1% in different parts of the world, etiology of this complex disease is not completely known. *HLA DQ2/8* haplotypes are the major predisposing genetic factors of CD, which is corroborated by twin studies and several GWAS studies^2,3^. GWAS on CD identified a total of 74 SNPs from 56 non-HLA loci, of which estimated 28 loci potentially alter the immune-related direct response that underlies the CD pathogenesis while several others regulate various associated biological processes and pathways. Notably, known 16 SNPs (~20%) associated with CD localized within or near to the genes with receptor function. This potentially highlighted the substantial contribution of receptor genes in CD.

The dietary gluten upon digestion is broken down into smaller proteins (glutenin and gliadin), which are absorbed in the small intestine and might trigger some abrupt cell signaling that initiate CD pathogenesis cascade. Cell-cell communication occurs via cascade of mechanisms including activation of receptors, transducers and enzymes. In gastrointestinal (GI) tissue smooth muscle contraction initiates with Ca^2+^ influx via L-type Ca^2+^ channels, which are activated through G-protein coupled receptors (GPCRs)^4,5^. Ca^2+^ sensitivity leads to inflammation induced hypocontractility and increased expression of cytokines. Cytokines binding to their receptors affects intracellular signaling and expression of GPCR, which can influence Ca^2+^ sensitization. However, in case of clinical inflammatory bowel disease (IBD) and experimental models of colitis, cytokines generated by Th17 cells are prominent in microbial infections, ischemia/reperfusion injury^5^. Notably, IL15, an unique cytokine is up regulated by stress and inflammation on any cell type but its expression requires binding with IL15R^6^. Another receptor NOTCH4 has been identified to stimulate NFkB signaling and inhibits E-cadherin, which is critical for gut epithelial biology^7^.

Existing molecular evidences and recent genetic studies highlighted the significant contribution of receptor genes in CD pathogenesis. Further genetic studies are warranted to identify and/or replicate receptor genes, which may help in identifying the environmental triggers and their cross talk with the cellular components for the onset of CD. In the present study we performed a hypothesis driven association study to identify novel receptor gene(s). Immunochip genotype data were available in-house for a total of 1227 north Indian subjects including 497 CD patients and 736 healthy controls^8,9^. Replication of identified risk variants was done using a European CD association summary statistics data, available to us. This study identified six novel risk variants from receptor genes, which are functionally relevant in CD pathogenesis. Based on the findings of this study a molecular model has been proposed which demonstrate the synergistic contribution of the identified novel genes. Several known therapeutic molecules were identified, which may be screened and tested in drug trials for their effectiveness in treating CD.

## Methods

### Strategy to select gene of interest

A manually curated  exhaustive list of receptor genes was made. Receptors were defined as known cellular protein that can bind to a specific ligand and initiate specific signaling cascade. KEGG and Gene Ontology (GO) pathway databases were used to curate a list of known receptor genes for his study^10,11^. Irrespective of their site of expression, pathways, cellular localization, all the genes with receptor functions were screened. Gene functions were confirmed by NCBI-Gene and RCSB-PDB (PDB). Alternative gene names and putative gene ids were also cross-checked in NCBI-Gene and Ensembl to avoid the possibility   of  any gene of interest to be missed/go unnoticed. Further, to reconfirm and validate their molecular and cellular function Uniprot and Gene Cards databases were used^12,13^.

### Genotype data and association statistics

Previously published association summary statistics of north Indian celiac disease study (using Illumina Immunochip platform) was available in-house. This dataset was used to identify novel receptor genes associated with CD. Majority of CD association studies were conducted  among groups with European ancestry, therefore, we replicated the identified CD associated risk variants in a published European association study dataset. A Dutch CD association study genotypes were obtained from Prof. Cisca Wijmenga’s laboratory, University Medical Center, Groningen, The Netherlands. Details of the population and genotype data characteristics are available elsewhere^8,14^.

Illumina Immunochip annotation file (hg19) was used to systematically pull out genetic variations (SNPs) mapped against all the receptor genes enlisted in the previously described exhaustive list of receptor genes. These genetic markers include exonic, intronic, UTRs and intergenic variations falling within the index genes as given in the Immunochip annotation file. Genomic coordinates of each of these markers were converted as per hg38 using UCSC LiftOver browser. Marker ids were upgraded as given in the 1000 Genomes database.

Summary statistics comprising of reference/minor alleles, minor allele frequency (MAF), association p-value and odds ratio (OR) with 95% CI were documented for each of the SNPs. For further analyses we selected all the SNPs with association p-value < 0.005, having minor allele frequency >15% to obtain approximately 90% study power to detect true associations. Level of significance was kept modest to identify and investigate the cumulative functions of biologically significant receptor genes and reduce chance to miss out any.

Association signals identified among north Indian  population were tested for cross-ethnic replication in a European celiac disease association study dataset mentioned above^8,14^. Associations were evaluated for SNPs originally identified in north Indians population. Conventional 5% level of significance was used to determine the replication of each of these SNPs.

To know more about the importance of identified genes in human diseases biology, cross-disease association was checked by scanning all the GWAS reports till date. Relevant data were extracted from NHGRI-EBI GWAS catalog (https://www.ebi.ac.uk/gwas/), which is an up-to-date public database to access and scan all the reported GWAS.

### *In-silico* functional annotation and gene prioritization

Genetic annotation of SNPs was done using RefSeq references. Molecular functions of each of these genes were obtained from UniProtKB/Swiss-Prot database^12^. Regulatory significance of these SNPs was checked on RegulomeDB and ENCODE database^15–17^. Based on their genetic location SNPs were evaluated for their putative contribution in epigenetic modifications and transcription factor binding. These include sites for DNase1 hypersensitivity, chromatin remodeling, histone modification, histone methylation (H3K4me1/3), acetylation (H3K27ac) and CTCF binding. From ENCODE, tissue specific epigenetic regulation data were evaluated for T-cell, B-cell and small intestine only, which are directly involved in the CD pathogenesis.

GTEx portal v7 (https://gtexportal.org) was used to investigate QTL properties of the SNPs^18^. Single-tissue eQTL was evaluated for each of the SNPs to know their functional implications. Emphasis was given for whole blood and small intestine because of their direct relevance in CD pathology.

### Identification of molecular interactions and networks

Biological relevance of identified genes was estimated by evaluating protein-protein interaction (PPI) and molecular networks. PPI analysis was performed using STRING (v11.1) database^19^. STRING is a powerful tool that extracts experimental or biochemical data from DIP, BioGrid, HPRD, IntAct, MINT, RCSB-PDB and curated data from Biocarta, BioCyc, Gene Ontology, KEGG and Reactome. Visual inspection of the protein-protein interactions and PPI confidence score were evaluated to understand the molecular cross-talk. All the genes from CD GWAS and identified genes in this study were used together to see the cumulative PPI. FDR p-value was considered to identify significant molecular interactions. 

Further, NetworkAnalyst 3.0 online tool was used for network enrichment analysis, generic PPI, and tissue specific co-expression to investigate the cumulative functional implication of the identified genes^20^. NetworkAnalyst is based on integration of machine learning and Walktrap algorithms^21^. Gene set enrichment analysis (GSEA) was performed to highlight molecular pathways, which are overrepresented by the genes identified in this study. Pathways curated in KEGG, Reactome, Gene Ontology (BP, CC and MF) and PANTHER (BP, CC and MF) databases were used for this analysis. Generic PPI was evaluated using non-redundant set of protein interactions curated in IMEx Interactome^22^. Tissue specific co-expression was studied  using functionally clustered genes in relevant tissues, such as in small intestine and whole blood. Adjusted p-value (based on permutation analysis and multiple corrections) was considered to identify significant molecular interactions.

### Drug target analysis

Replicated genes were accessed for possible interaction(s) with known drugs, either approved for human use or under investigation. DrugBank (v5.1.2, released 2018-12-20) database^23^ was used for this analysis. This database presently has a total of 12,110 drug entries (including 2,554 approved small molecule drugs, 1,280 approved proteins/peptide drugs, 130 nutraceuticals and over 5,842 experimental compounds) and 5156 non-redundant proteins against query target(s).

## Results

### Gene list and association

Dense genotype data for 269 receptor genes were available for the study. 5431 genetic variations were assessed for their association. For north Indian population, association results of 1227 individuals, including 497 cases and 736 controls were available^8^. Six novel genes were found associated (p ≤ 0.005) with CD in north Indian population (Table 1). Only two markers, rs29231 (p = 1.21e-11) from *MOG* and rs3025643 (p = 1.6e-7) from *GABBR1* were identified as statistically significant following multiple corrections. Other four SNPs from *OR2H2*, *ABCF1*, *ADRA1A*, and *ACVR2A* were identified with suggestive p values. Top four SNPs from *MOG*, *GABBR1*, *OR2H2* and *ABCF1* are localized within extended MHC locus. Pair-wide LD (r^2^) estimation showed absence of detectable LD between these SNPs (from MHC region) and known *HLA-DQ2* and *HLA-DQ8* alleles or their representative SNPs. Only one SNP, rs29231 from *MOG* showed moderate LD (r^2^ < 0.5) with *HLA-F*, *HLA-A* and *HLA-G* among Asians only. Two non-HLA SNPs, rs10102024 and rs7560426 were identified from *ADRA1A* and *ACVR2A*. These are novel CD associated loci and 50 kb windows on both sides of these SNPs did not overlap with any reported CD associated loci.

**Table 1:** Association statistics and replication status of six CD associated SNPs identified among north Indians population in the present study.

Markers	Allele	Chr_bp (hg38)	Location	Mapped Genes	North Indian			European (Dutch)		
					MAF	P-value	O.R	MAF	P-value	O.R
rs29231	A	6:29650748	6.2 kb 5′ *MOG*	*MOG*	0.16	1.21e-11	2.16 (1.73–2.70)	0.14	0.43	0.93 (0.78–1.11)
rs3025643	T	6:29602178	48 bp 3′ *GABBR1*	*GABBR1*	0.21	1.60e-07	1.73 (1.41–2.12)	0.29	5.38e-08	0.68 (0.59–0.78)
rs1233388	C	6:29585950	2 kb 5′ *OR2H2*	*OR2H2*	0.31	0.0002	0.69 (0.56–0.83)	0.20	3.29e-05	0.72 (0.61–0.84)
rs9262119	T	6:30595773	4.2 kb 3′ *ABCF1*	*ABCF1*	0.27	0.0005	1.41 (1.16–1.70)	0.07	0.0002	0.62 (0.48–0.79)
rs10102024	C	8:26927854	61 kb 5′ flanking *ADRA1A*	*ADRA1A*	0.26	0.003	1.34 (1.10–1.63)	0.11	0.56	1.06 (0.87–1.28)
rs7560426	G	2:147466175	37.9 kb 5′ flanking *ACVR2A*	*ACVR2A*	0.16	0.004	0.69 (0.53–0.89)	0.06	0.51	1.09 (0.85–1.39)

Positional annotation of the SNPs was recorded from RefSeq and dbSNP.

All six SNPs were tested for cross-ethnic replication in a European celiac disease association statistics. Originally 1150 CD subjects and 1173 controls were used for the Dutch association study using Illumina Immunochip platform^8^. Three SNPs rs3025643 from *GABBR1*, rs1233388 from *OR2H2* and rs9262119 from *ABCF1* were replicated (p ≤ 0.05) (Table 1). Most significant association signal from north Indian population rs29231 did not replicate in Europeans (Dutch), however strong association (p = 5.88e-33, OR = 2.37) was observed for a neighboring SNP rs3129073 (D’ = 1, r2 = 0.05).

Except for  *OR2H2*, six other genes were previously reported in GWAS for their association with other diseases. More than one SNP were previously identified with genome-wide significant p-values for each of these six genes. Several immune mediated and inflammatory diseases were found associated with these genes (Supplementary Table 1).   

### Performance of functional annotations of the associated SNPs

Top four associated SNPs (rs29231, rs3025643, rs1233388, and rs9262119) localized very close (<6.5 kb) to the upstream or downstream of the associated genes, which overlaps with the regulatory regions that control the gene expression or mRNA stability (Table 1). Identified genes have considerable expressions in whole blood, lymph nodes and small intestine and have relevant functional implications that could affect inflammatory diseases such as celiac disease (Supplementary Table 2).

Evidences from RegulomeDB have shown that, rs29231 from *MOG* has a strong role in regulating the epigenetic modifications at the 5′ flanking sequences of the gene (Supplementary Table 2). Further analysis focused on tissue specific significant epigenetic signatures at the identified SNPs in T-cells, B-cells and small intestine was insightful. Evidences from ENCODE database showed that the DNA sequence around rs29231 has significant signatures for very high degree of DNase I hypersensitivity, histone methylation (H3K4me1/3), histone acetylation (H3K23ac) and zinc finger transcription factors (CTCF) binding. Such strong and significant transcriptional regulation signatures were found for rs9262119 from *ABCF1*, and rs7560426 from *ACVR2A* (Supplementary Table 3). Single tissue eQTL data  were available for five SNPs in GTEx portal, where all the respective genes are expressed in all or at least one of the relevant tissues (whole blood and small intestine). Evidences of strong eQTL  were observed from rs29231 (*MOG*), rs3025643 (*GABBR1*), rs1233388 (*OR2H2*) and rs9262119 (*ABCF1*). These four SNPs were found to have significant eQTL effects on genes including *IFITM4P*, *HLA-F*, *ZFP57*, *HLA-J*, *HLA-A*, *MICD*, *RPL23AP1*, *HCG4P5*, *TRIM27* and *IER3* in whole blood tissue and *IFITM4P* and *HCG4* in small intestine (Supplementary Table 2).

### Pathway enrichment analysis

All the six novel associated genes (Table 1) and genes with strong eQTL (Supplementary Table 2) were tested for their enrichment in curated pathways from different databases. Pathway enrichment analysis identified to be significantly associated (adjusted P_adj_ ≤ 0.05) with key cellular pathways such as, Receptor activity (P_adj_ = 6.5e-4), Transmembrane signaling (P_adj_ = 0.0002), and Endosomal/vacuolar pathway (P_adj_ = 0.04). Cellular components such as Integral/intrinsic plasma membrane (P_adj_ = 0.02) was found enriched (Supplementary Table 4).

### Identification of protein-protein interactions (PPI)

**Figure 1:**
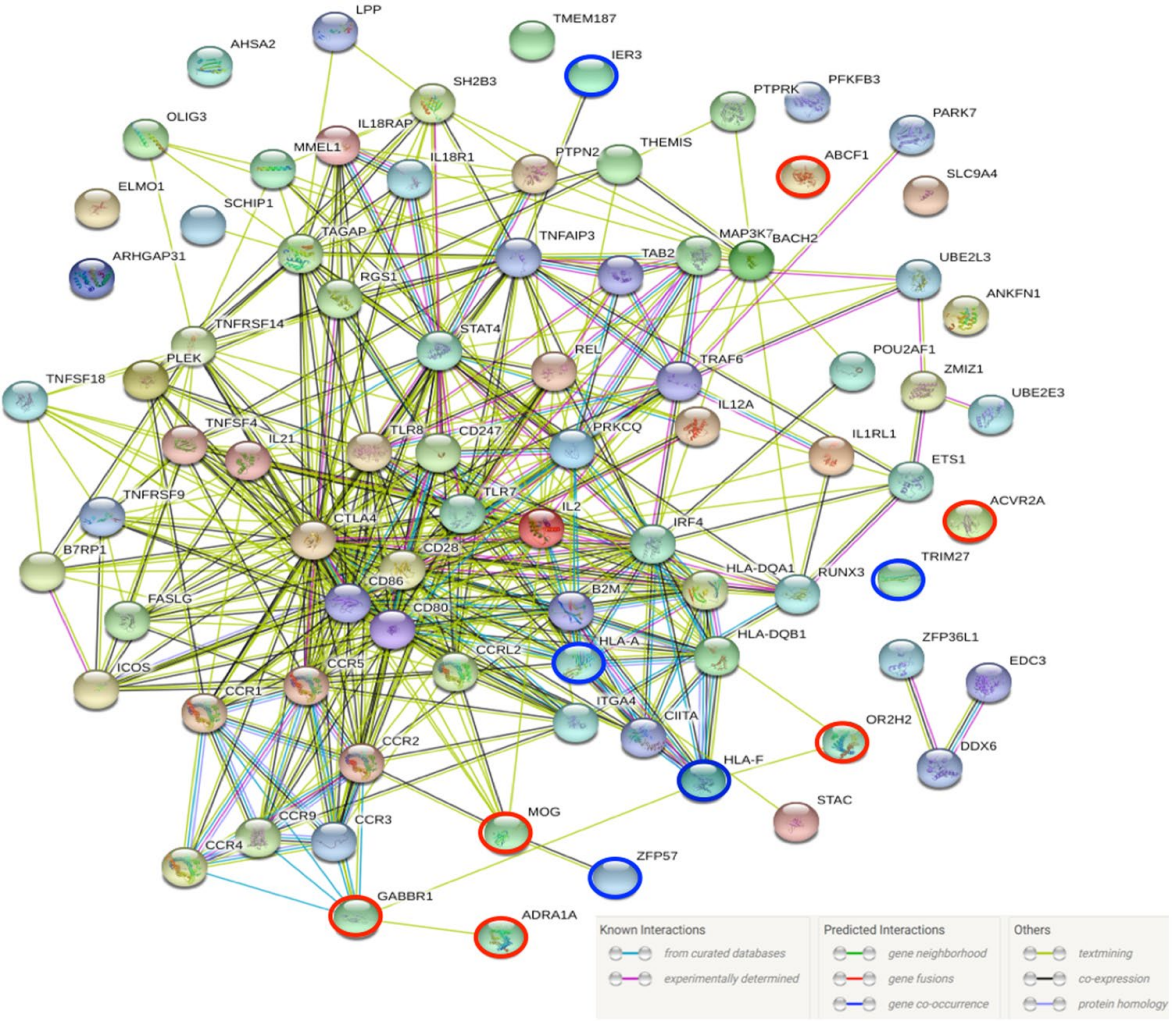
Functional PPI network (PPI enrichment p-value < 1.0e-16) between GWAS reported known CD genes and genes identified in this study. Red circled genes were identified in this study (Table 1) and blue circled genes were identified through tissue eQTL (Supplementary Table 2). Predicted functional partners identified are EDC3, TAB2, B2M, TRAF6 and CD86 with Score > 0.99.

 To elucidate the collective contribution of significantly associated receptor genes (Table 1) PPI-networks were reconstructed and evaluated. Generic PPI reconstructed using NetworkAnalyst 3.0 showed *MOG*, *GABBR1*, *OR2H2*, *ABCF1*, *ADRA1A*, and *ACVR2A* nodal proteins involved in different clusters of protein-protein interactions. Through direct and indirect interactions, we identified protein clusters enriched in key interactions such as, KEGG_TGF-beta signaling pathway (P_adj_ = 1.6e-6), Protein processing in ER (P_adj_ = 4.6e-6), Antigen processing: ubiquitination & proteosome degradation (P_adj_ = 2.13e-17), ER phagosome pathway (P_adj_ = 4.83e-9), TRIF-mediated TLR3/TLR4 signaling (P_adj_ = 2e-5), Cellular protein catabolic process (P_adj_ = 6.31e-18) among several others (Supplementary Table 4). PPI-Network reconstructed using STRING v11.0 showed molecular cross talk between five out of six genes identified in this study with all other known CD associated genes. *MOG*, *GABBR1*, *ADRA1A*and *OR2H2* were found in a common functional PPI network (Fig. 1).

### Co-expression in target tissues

Tissue specific co-expression profiling were evaluated for small intestine and whole blood using NetworkAnalyst tool. Co-expressed genes in small intestine are enriched for inflammatory pathways such as Regulation of T cell activation (P_adj_ = 1.54e-10), Regulation of lymphoid activation (P_adj_ = 5.78e-9), Immunomodulatory interactions between a lymphoid and a non-lymphoid cell (P_adj_ = 2.11e-6), and TCR signaling (P_adj_ = 1.4e-5) among several others. On the other hand, several transcription and translation associated pathways, such as RNA binding (P_adj_ = 4.41e-15), Ribonucleoprotein complex biogenesis (P_adj_ = 0.002) and tRNA aminoacylation (P_adj_ = 0.003) among several others were found enriched due to co-expression of key regulatory genes in whole blood (Supplementary Table 4).

### Identification of potential drug targets

To identify potentially new therapeutic targets for CD, we investigated six identified genes. Several drugs/therapeutic compounds were identified which are either approved or under investigation against *GABBR1* and *ADRA1A*. Five therapeutically potential compounds/drugs were found, which are approved by FDA and used to treat chronic and inflammatory diseases/conditions (Supplementary Table 5).

## Discussion

High-throughput genomic studies and meta-analysis replicated classical *HLA* alleles (*HLA-DQ2/8*) and identified 56 non-HLA loci for CD. Majority of these studies were done on populations with European ancestry and could address only 55% of the disease heritability. In this hypothesis driven comprehensive study we attempted to look beyond statistically significant genes and identified genes with functional relevance. We performed this study on genetically distinct north Indian population and identified novel and/or population specific risk variant(s). Present study identified functionally relevant six novel susceptible receptor genes among north Indians and rationalized their synergistic role in CD. Among these, three genes namely, *GABBR1*, *OR2H2* and *ABCF1* were replicated in the European dataset, and *MOG*, *ADRA1A* and *ACVR2A* were found north Indian specific (Table 1).

Myelin oligodendrocyte glycoprotein (*MOG*) and gamma-aminobutyric acid type B receptor subunit 1 (*GABBR1*) were identified with statistically significant p values (p < 5e-7). Top signal from *MOG*, rs29231 could not get replicated in Europeans. Other significant association signal rs3129073 from *MOG*, which are not in LD (D’ = 1, r2 = 0.05) with the reported SNP, are replicated in Europeans, which indicated that this non-replication could be due to ethnicity specific LD background between north Indians and Europeans. Top four SNPs are located in the extended MHC region, however they do not share detectable LD between them and with other HLA markers, especially with known CD associated HLA markers, which indicates their independent association with CD. Though these genes are novel for CD, except *OR2H2* other five genes were reported in recent GWAS and known to be associated with several anthropometric traits and inflammatory diseases including lung cancer, lung adenocarcinoma, mumps, leukocyte count, Graves’ disease, breast cancer, multiple sclerosisetc. (Supplementary Table 1)^24–28^. Such cross-disease associations may indicate: (a) phenotypic and functional significance of the identified genes, and (b) presence of shared pathways and molecular functions, which could be used to improve the disease management and design novel therapeutics.

It was noteworthy that, top four SNPs are located near to the coding sequences of the gene and have very strong regulatory significance in small intestine, T cells and B cells (Supplementary 2 and 3), which suggests their direct functional implication in CD. Further, identified markers from *MOG*, *GABBR1*, *OR2H2* and *ABCF1* were found to have significant eQTL effects on neighboring genes in whole blood and small intestine (Supplementary Table 2). Identified genes were shown enriched in pathways that are relevant for regulated cellular phagocytosis and protein metabolism^29,30^. Collective contribution of the six novel genes identified in this study along with already reported CD genes was identified for the pathogenesis of CD (Fig. 1). This inclusive network also includes genes, which shows strong eQTL (in whole blood and small intestine) with identified six novel genes. Together these findings showed the relevance of the identified novel genes in accordance with the previous findings. Identified genes in this study were found to interact with their partners and influence immune functioning, key molecular signaling (TGF-beta, TLRs), protein metabolism, endoplasmic reticulum functioning protein processing, cellular adhesion in general as well as in small intestinal cells and whole blood cells (Supplementary Table 4). These together highlighted functional involvement of novel genes identified in this study in CD.

*MOG* encodes a membrane glycoprotein expressed on the oligodendrocyte cell surface and the outermost surface of myelin sheaths. It plays significant role in T-cell receptor signaling pathway, cell adhesion and regulation of immune response. The functional status of *MOG* depends on its glycosylation, which maintains CLRs/TLRs balance. *MOG* keeps the antigen presenting cells in central nervous system (microglia) of draining lymph nodes (dendritic cells) in an immature state. APCs degrade the antigen and presents degraded peptide via MHC class II to CD4+ T cells. Thus, any alteration in the N linked glycosylation of *MOG* can lead to auto-aggressive T cell activation as well as leading to amplification of local presentation of myelin antigens, (re-) activation of T cells and influx of additional cell types and soluble factors into the CNS, contributing to Multiple Sclerosis (MS) and other autoimmune pathology^30^. Our study identified rs29231, located at immediate upstream of *MOG* and potentially can regulate expression of several neighboring genes in T-cells, B-cells and small intestine through epigenetic modifications (Supplementary Table 2, 3). rs29231 has strong eQTL effect of *ZFP57* expression, which is known transcriptional repressor that regulates expression of *IGF2*, and associated with transient neonatal diabetes mellitus type 1 (TNDM1)^31,32^.

*GABBR1* and *ABCF1* are the other interacting proteins that have shown to have significant role in regulating gene expression through alteration of epigenetic signatures in small intestine and immune cells (Supplementary Figs 2 and 3). *GABBR1* encodes for gamma-aminobutyric acid (GABA), which is the main inhibitory neurotransmitter in the mammalian central nervous system. This receptor functions as a heterodimer with GABA(B) receptor 2 and downregulates p38 MAPK^33^. p38 MAPK along with TNF-α recruits pro-inflammatory agents like IL1, IL6 and MMP3. p38 MAPK releases prostaglandin and interacts with STAT4, ERKs, MKKs and NFkB that results in pathway of inflammatory disease^34^. Both *MOG* and *GABBR1* are integral component of plasma membrane. Interestingly, genome wide scan of Ashkenazi Jewish Crohns’s disease suggest *MOG* and *GABBR1* as novel susceptibility loci^35^. *ABCF1* is a member of the superfamily of ATP-binding cassette (ABC) transporters. ABC proteins transport various molecules across extra- and intra-cellular membranes. Unlike other members of the superfamily, this protein lacks the transmembrane domains, which are characteristic of most ABC transporters. It may be regulated by TNF-alpha and play a role in enhancement of protein synthesis and the inflammation process. Upon TNFα binding to both TNFR1 and TNFR2 expressed on smooth muscle activates NFkB, TLR2, and TLR4. In addition, NFκBfurther activates ICAM, MCP-1 and IL-8, which can be induced to generate inflammatory mediators as well as reactive oxygen species (ROS) that are proposed to contribute to reduce smooth muscle contractility^36,37^.

Another identified gene activin receptor type-2A (*ACVR2A*) encodes for receptor that mediates the functions of activins, TGF-β, and BMP. Expression of *ACVR2A* is required for the differentiation of T cells and specific only for CD4^+^ Th17 cell types. IL-17 secreted by Th17 cell types and is a key pro-inflammatory molecule associated with autoimmune diseases such as rheumatoid arthritis, IBD etc^38^. It highlights a common Th17 mediated pathogenesis mechanism for autoimmune gastrointestinal diseases, such as IBD and CD. Th17 cell differentiation and T cell activation leads to cryptic hyperplasia and villous atrophy which is a classical clinical phenotype in CD. *ACVR2A* thus play a critical role in autoimmune disease progression and could be evaluated for common therapeutic purpose.

**Figure 2:**
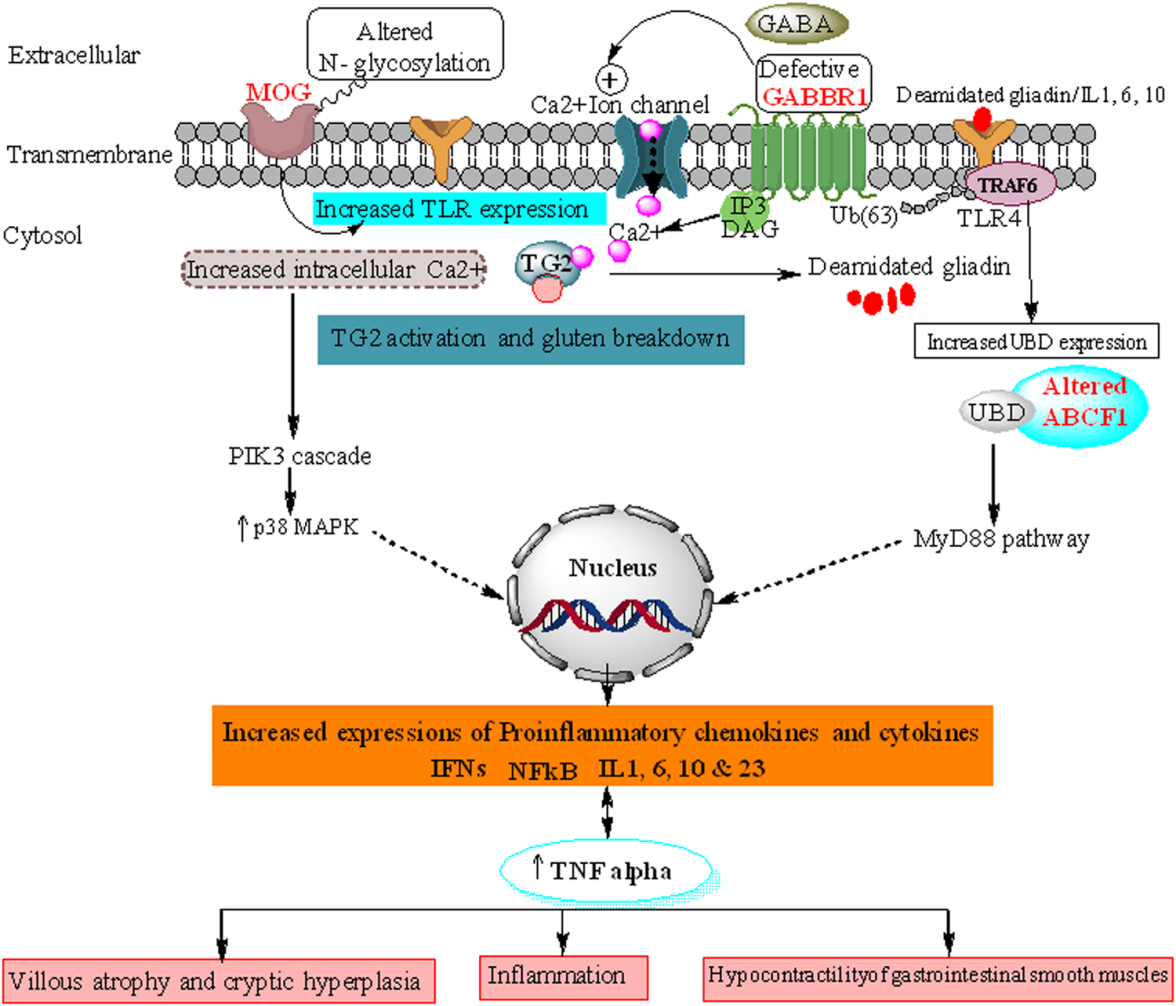
A suggestive model for receptor-mediated pathway involved in CD biology.

Based on the critical findings from this study we have suggested a model for CD pathology (Fig. 2). We proposed that individually and in synergy these genes regulate a cluster of biological processes and molecular functions that modify the cellular homeostasis, which may leads to pathogenesis of CD. This study identified *MOG*, which is a surface receptor and any alteration in its N- glycosylated chain leads to increased TLR expression which can bind to deaminated gliadin/interleukins 1, 6, 10 and 23 and increased expression of TLR4 has been found in intestinal epithelial of CD patients. *GABBR1* is a G- protein coupled receptor whose main function is to inactivate Ca^++^ ion channel. Altered *GABBR1* leads to increased intracellular Ca^++^ which results in activation of transglutaminase (TG2) and phosphoinositol kinase 3 (PIK3) cascade. Activated TG2 deamidates gluten to gliadin. Deamidated gliadin causes increased expression of UBD as well as TNF alpha. *ABCF1* being an conjugating enzyme of UBD leads to MyD88 dependent pathway resulting in the release of proinflammatory chemokines and cytokines which ultimately progresses to the major symptoms of celiac disease namely: villous atrophy and cryptic hyperplasia, inflammation and hypocontractility of GI smooth muscles (Fig. 2).

Hence, this association study identified a set of functionally related genes having promising contribution in inflammatory pathways and autoimmune conditions. Confirmatory functional genomics studies must be performed to establish the detailed functional implications of these genes in CD. Furthermore, the approved drugs or therapeutic compounds identified in this study are currently in use to treat other diseases, and could be tested to treat CD in a controlled trial.

## Conclusions

Present study identified six novel associated SNPs with convincing study power. These genes highlighted clusters of relevant molecular pathways, which are  critical for CD pathogenesis. Identified genes were previously reported for several inflammatory diseases, which directly advocated for their role in disease biology. Follow-up functional studies are warranted to confirm the involvement of these genes in CD.

## Supplementary information

Supplementary Informations

## Data Availability

Summary statistics data for north Indians and Dutch can be obtained from Dr. Sabyasachi Senapati, Central University of Punjab, India, and Prof. Cisca Wijmenga, UMCG, Groningen, The Netherlands respectively upon request.
