# Supplementary material for: Association study identified biologically relevant receptor genes with synergistic functions in celiac disease
Source: Sci Rep. 2019 Sep 25;9:13811. doi: 10.1038/s41598-019-50120-4 (PMC6761106; doi:10.1038/s41598-019-50120-4)
Supplement: Supplementary file 1 — Supplementary Informations [file 41598_2019_50120_MOESM1_ESM.pdf]

## Supplementary Information for:

# Association study identified biologically relevant receptor genes with synergistic functions in celiac disease

**Pratibha Banerjee[1], Sandilya Bhagavatula[1], Ajit Sood[2], Vandana Midha[3], Thelma BK[4], Sabyasachi Senapati[1]**

[1]Department of Human Genetics and Molecular Medicine, School of Health Sciences, central University of Punjab, Bathinda, Punjab, India
[2]Department of Gastroenterology, Dayanand Medical College & Hospital, Ludhiana, Punjab, India.
[3]Department of Medicine, Dayanand Medical College & Hospital, Ludhiana, Punjab, India.
[4]Department of Genetics, University of Delhi South Campus, New Delhi, India.

## Supplementary Tables: 1-5

**Supplementary Table 1:** List of GWAS reported traits associated with genes identified in this study. Traits with inflammatory components are underlined.

| Genes | GWAS associated traits | Study accession numbers |
|---|---|---|
| ***MOG*** | Plantar warts | GCST005005 |
| | Pulmonary function | GCST001784 |
| | Lung cancer | GCST004748 |
| | Tonsillectomy risk | GCST005014 |
| | Colorectal health | GCST005147 |
| | Tuberculosis | GCST005006 |
| | Autism spectrum disorder | GCST004521 |
| | Blood protein level | GCST004365 |
| | Lung adenocarcinoma | GCST004744 |
| ***GABBR1*** | Diastolic blood pressure | GCST006227 |
| | Neuroticism | GCST005232 |
| | Mumps | GCST005003 |
| | Unipolar depression | GCST005902 |
| | Leukocyte count | GCST004610 |
| | Schizophrenia | GCST003880 |
| | Nicotine dependence | GCST004294 |
| | Mood swings | GCST006944 |
| | Feeling miserable | GCST006943 |
| | Depressed effect | GCST006475 |
| | Ulna and radius bone mineral density | GCST006446 |
| ***ABCF1*** | Schizophrenia | GCST007201 |
| | General cognitive ability | GCST006269 |
| | Cold sores | GCST005000 |
| | Graves' disease | GCST001219 |
| | Breast cancer | GCST004988 |
| | Mild influenza (H1N1) infection | CGST003124 |
| ***ADRA1A*** | Blood metabolite levels | GCST002443 |
| | Reaction time | GCST006268 |
| | Treatment response to severe sepsis | GCST001402 |
| ***ACVR2A*** | Serum creatinine measurement | GCST003372 |
| | Neutrophil percentage of granulocytes | GCST004623 |
| | Feeling tense | GCST006952 |
| | Neuroticism | GCST005232 |
| | Feeling hurt | GCST006951 |
| | Educational attainment | GCST006571 |
| | Systolic blood pressure | GCST007087 |
| | Basophil count | GCST004618 |
| | Diastolic blood pressure | GCST006627 |

**Supplementary Table 2:** Functional annotation of associated SNPs identified in the study. Data mining was done from RegulomeDB, GTEx and GeneCards.

*Significant score given by RegulomeDB (1f ).

# Functional data for rs3129073 (D'=1) was given, as proxy for rs29231.

| Markers | Gene name | RegulomeDB Overall Score | Tissue specific eQTl | | | Molecular Functions |
|---|---|---|---|---|---|---|
| | | | Gene name | Whole blood (p value) | Small Intestine (p value) | |
| rs29231[#] | *MOG* | **1f*** | *IFITM4P*<br>*HLA-F*<br>*ZFP57*<br>*HLA-J*<br>*HLA-A*<br>*MICD*<br>*RPL23AP1*<br>*HCG4P5*<br>*HCG4* | 4.2e-15<br>1.4e-10<br>1.6e-10<br>6.0e-08<br>2.2e-06<br>3.9e-06<br>7.0e-06<br>2.0e-05<br>-- | 8.0e-08<br>--<br>--<br>--<br>--<br>--<br>--<br>--<br>2.0e-06 | Myelin oligodendrocyte glycoprotein directly involved in immune-mediated demyelination of oligodendrocytes. Involved in pathways for neural stem cell differentiation. Mediate cell-cell communication. Acts as a receptor for rubella virus. Have ubiquitous expression including small intestine, WBCs and lymph nodes. |
| rs3025643 | *GABBR1* | 5 | *TRIM27* | 5.0e-05 | -- | Gamma-aminobutyric acid type B receptor subunit 1 encodes GABA receptor in the CNS. Involves in G-protein coupled receptor activity. Peptide ligand-binding receptors and G- protein coupled GABA receptor activation. Considerable expression is seen in WBCs, lymph nodes and Small intestine. |
| rs1233388 | *OR2H2* | 6 | *TRIM27* | 9.7e-07 | -- | Olfactory receptor family 2 subfamily H member 2 is GPCR family protein. Helps in G protein-coupled receptor activity and olfactory responses. Limited expression in small intestine. |
| rs9262119 | *ABCF1* | 5 | *IER3* | 3.0e-05 | -- | ATP binding cassette subfamily F member 1 is a membrane bound receptor. It promotes CDK-mediated phosphorylation and removal of Cdc6 and transport of glucose and other sugars, bile salts and organic acids, metal ions and amine compounds |
| rs10102024 | *ADRA1A* | No Data | -- | -- | -- | Adrenoceptor alpha 1A is a member of G protein-coupled receptor superfamily. It activates a phosphatidylinositol-calcium second messenger system. Considerable expression is seen in WBCs, lymph nodes and Small intestine. |
| rs7560426 | *ACVR2A* | 6 | -- | -- | -- | Activin A receptor type 2A is a cell membrane bound receptor, mediates the functions of activin, a TGF-beta superfamily protein. It has protein serine/threonine kinase activity. Considerable expression is seen in WBCs, lymph nodes and Small intestine. |

**Supplementary Table 3:** Tissue specific functional significance of associated SNPs, based on ENCODE database. Data were evaluated for functionally implicated cell types, i.e, T-cells, B-cells and small intestine. Score value >1.64 was considered as significant and was highlighted with green (DNase I hypersensitivity), red (methylation), yellow (acetylation) and blue (CTCF binding).

| Markers | Gene name | T-cell | | | | B-cell | | | | Small intestine | | | |
|---|---|---|---|---|---|---|---|---|---|---|---|---|---|
| | | DHS | Methylation | Acetylation | CTCF | DHS | Methylation | Acetylation | CTCF | DHS | Methylation | Acetylation | CTCF |
| rs29231 | *MOG* | | | | -- | | | | | | | | -- |
| rs3025643 | *GABBR1* | 1.06 | -0.13 | 0.8 | -- | -- | 0.86 | 0.85 | 0.17 | 0.97 | -0.26 | -1.08 | -- |
| rs1233388 | *OR2H2* | -- | -- | -- | -- | -- | -- | -- | -- | -- | -- | -- | -- |
| rs9262119 | *ABCF1* | | 0.44 | | -- | | -0.52 | 0.64 | | | 0.53 | 0.69 | -- |
| rs10102024 | *ADRA1A* | -- | -- | -- | -- | -- | -- | -- | -- | -- | -- | -- | -- |
| rs7560426 | *ACVR2A* | 0.86 | -0.06 | 0.12 | -- | | 0.22 | 0.00 | -0.23 | | 0.54 | | -- |

**Supplementary Table 4**: Significant results of pathway enrichment analysis, protein-protein interaction analysis and co-expression (whole blood and small intestine). Data was collected from KEGG, Reactome, Gene Ontology and Panther databases. Where BP= Biological Process, CC: Cellular Component, and MF: Molecular Function.

| **Database** | **Pathway Enrichment Analysis** | **Adjusted p- value** |
|---|---|---|
| REACTOME | Endosomal/Vacuolar pathway | 0.04 |
| GO:MF | Transmembrane signaling<br>Receptor activity | 0.0002<br>6.5e-4 |
| GO:CC | Integral/intrinsic plasma membrane | 0.02 |
| **Generic PPI Analysis** | | |
| KEGG | TGF-beta signaling pathway<br>Protein processing in ER<br>Antigen processing and presentation<br>T cell receptor signaling pathway | 1.6e-6<br>4e-6<br>0.001<br>0.005 |
| REACTOME | Class I MHC mediated antigen presentation<br>Adaptive immune system<br>Antigen processing: ubiquitination & proteosome degradation<br>ER-phagosome pathway<br>TRIF-mediated TLR3/TLR4 signaling<br>Signaling by activin | 1e-23<br>1.41e-20<br>2.13e-17<br>4.83e-9<br>2e-5<br>0.0002 |
| GO:BP | Macromolecule modification<br>Protein modification by small protein conjugation<br>Cellular protein catabolic process<br>Protein polyubiquitination | 3.17e-18<br>4.37e-18<br>6.31e-18<br>5.06e-16 |
| GO:MF | Small conjugating protein ligase activity<br>Amino acid ligase activity<br>Receptor signaling protein activity | 1.06e-22<br>1.06e-22<br>6.93e-6 |
| PANTHER:BP | Viral process | 2.69e-12 |
| PANTHER:MF | Ubiquitin-protein transferase activity<br>Transmembrane receptor protein serine/threonine kinase activity | 1.39e-17<br>1.27e-5 |
| **Small Intestine specific co-expression** | | |
| KEGG | T cell receptor signaling pathway<br>Cell adhesion molecules (CAMs) | 0.0008<br>0.0008 |
| REACTOME | Immunoregulatory interactions between a lymphoid and a non-lymphoid cell<br>TCR signaling | 2.11e-6<br>1.4e-5 |
| GO:BP | Regulation of T cell activation<br>Regulation of lymphoid activation | 1.54e-10<br>5.78e-9 |
| PANTHER:BP | Immune system processing | 0.0008 |
| **Whole Blood specific co-expression** | | |
| REACTOME | RNA polymerase II pre-transcription events | 0.003 |
| | tRNA aminoacylation | 0.003 |
| GO:BP | Ribonucleoprotein complex biogenesis | 0.002 |
| GO:MF | Ligase activity, forming carbon-oxygen bonds | 0.004 |
| PANTHER:BP | Translation | 0.004 |
| PANTHER:MF | RNA binding | 4.41e-15 |

**Supplementary Table 5**: Potential drugs targets and its action (Drug bank).

| Identified genes | Targeted drug | Drug Group | Pharmacological action | Indication |
|---|---|---|---|---|
| *GABBR1* | Baclofen | Approved | Agonist | Multiple sclerosis |
| | Progabide | Experimental | Agonist | Epilepsy |
| | Tezampanel | Investigational | AMPA antagonists & inhibitors | Pain (acute or chronic), migraine and cluster headaches. |
| | Vigabatrin | Approved | Agonist | Resistant epilepsy, refractory complex partial seizures |
| | Arbaclofen | Investigational | Agonist | Multiple sclerosis, autism and fragile X syndrome |
| | ArbaclofenPlacar bil | Investigational | Agonist | Multiple sclerosis, acute back spasms, and GERD |
| | Gabapentin | Approved, Investigational | $Ca^{+2}$ channel blockers | Epilepsy |
| | Taurine | Approved, Nutraceutical | Agonist | Treat negative nitrogen balance in pediatric patients, impaired gastrointestinal absorption or protein requirements are substantially increased |
| *ADRA1A* | Labelatol | Approved | Antagonist | Hypertension, Stroke, intracerebralhemorrhage |
| | Dobutamine | Approved | Agonist | Cardiac decompensation,Coronary Artery Disease |
